# (−)‐Epigallocatechin Gallate Ameliorates Chronic Stress‐Induced Depression‐Like Behavior in Rats by Inhibiting the NF‐κB/Caspase‐1 Signaling Pathway and Reducing IL‐1β and IL‐18 Expression

**DOI:** 10.1002/fsn3.70961

**Published:** 2025-09-26

**Authors:** Guixian Wu, Jieyu Xu, Wanhua Wu, Jian Jin, Shanqian Li, Yue Huang, Zongyi Zhang, Yue Hu, Yulin Zhang, Yuling Liu, Yao Xu, Meimei Zheng, Chaozhi Xu, Wenjun Lu, Hongxian Wu, Lina Hu

**Affiliations:** ^1^ School of Public Health Guilin Medical University Guilin China; ^2^ Guangxi Key Laboratory of Environmental Exposomics and Whole Life Cycle Health Guilin China; ^3^ Key Cultivation Laboratory of Life Cycle Health Care Research Guilin China; ^4^ Guilin University of Aerospace Technology Guilin China; ^5^ Department of Clinical Laboratory Guilin Social Welfare Hospital Guilin China; ^6^ Nutrition Department of Liuzhou Workers' Hospital Liuzhou China; ^7^ Department of Pediatrics The First Affiliated Hospital of Guangxi Medical University Nanning China; ^8^ Communicable Disease Control Branch Qingdao City Center for Disease Control and Prevention Qingdao China; ^9^ Institute of Drug Inspection Technology Shanxi Inspection and Testing Center Taiyuan China; ^10^ Department of Nutrition Second People's Hospital of Ya'an City Ya'an City China; ^11^ Medical Information Management, School of Humanities and Management Guilin Medical University Guilin China; ^12^ General Practice Department Affiliated Hospital of Guilin Medical University Guilin China; ^13^ Department of Cardiology, Zhongshan Hospital Fudan University, Shanghai Institute of Cardiovascular Diseases Shanghai China; ^14^ State Key Laboratory of Cardiovascular Diseases, Zhongshan Hospital Fudan University Shanghai China; ^15^ NHC Key Laboratory of Ischemic Heart Diseases Shanghai China; ^16^ Key Laboratory of Viral Heart Diseases Chinese Academy of Medical Sciences Shanghai China; ^17^ National Clinical Research Center for Interventional Medicine Shanghai China

**Keywords:** chronic stress, depression‐like behaviors, EGCG, pyroptosis

## Abstract

This study aimed to further clarify the ameliorative effects of epigallocatechin‐3‐gallate (EGCG) on depression and depression‐like behaviors induced by chronic unpredictable mild stress (CUMS) in female Sprague–Dawley (SD) rats. EGCG was administered via intraperitoneal injection, and liver function and blood lipid profiles were assessed. Western blot analysis was employed to determine the expression levels of caspase‐1 and nuclear factor kappa B (NF‐κB), as well as inflammatory factors interleukin‐1β (IL‐1β) and interleukin‐18 (IL‐18). The results showed that CUMS induced depression‐like behaviors (including anhedonia and behavioral despair) in rats, accompanied by reduced body weight, decreased liver index, and dyslipidemia. Western blot analysis revealed significantly elevated expression of pyroptosis‐related proteins (NF‐κB, cleaved‐caspase‐1, IL‐1β, and IL‐18) in both the liver and brain tissues of the CUMS group. EGCG treatment could reverse these results. Notably, female rats exhibited more severe CUMS‐induced depression‐like behaviors than male rats. These findings suggest that EGCG can alleviate CUMS‐induced depression‐like behaviors (anhedonia and behavioral despair) in female rats. This protective effect may be mediated by inhibiting the NF‐κB/caspase‐1 pyroptosis pathway, reducing IL‐1β and IL‐18 expression, thereby improving hepatocellular pyroptosis and alleviating liver injury.

## Graphical abstract

Under chronic stress, female rats are more susceptible to developing depression compared to male rats. EGCG inhibits CUMS‐induced pyroptosis in rat liver and brain tissue by inhibiting the activation of the NF‐кB/caspase‐1 signaling pathway, reducing liver damage, and improving depression‐like behaviors in rats.

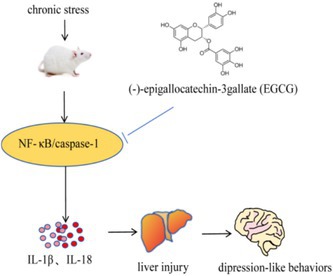

## 1 Introduction

Depression is a chronic mental disorder characterized by mood disturbances, impaired thinking, anxiety, cognitive/social dysfunction, and is recognized as one of the most pressing global mental health issues (Monroe and Harkness 2022). It can affect anyone, with individuals exposed to abuse, significant losses, or other stressful events being more vulnerable. Epidemiological data indicate that women are more susceptible to depression than men (Bhurtyal et al. 2022): 3.8% of the global population (approximately 280 million people) suffer from depression, including 5% of adults (4% men and 6% women), and 5.7% of adults aged 60 and over (Dodd et al. 2023). The prevalence of depression in women is approximately 50% higher than in men, and over 10% of pregnant and postpartum women are affected globally (Woody et al. 2017). Since the COVID‐19 pandemic, the number of new cases of major depression has increased significantly in 2020 (a 27.6% increase from previous years), imposing heavy financial and emotional burdens on patients and their families (COVID‐19 Mental Disorders Collaborators 2021). Despite clear gender differences in depression reported clinically, the underlying mechanisms remain poorly understood.

Chronic stress (e.g., occupational or academic stress) is a key risk factor for depression, inducing sustained inflammatory responses that promote the release of pro‐inflammatory cells and cytokines. This can contribute to arteriosclerosis, cardiovascular disease, and increased risk of cancer and tumor metastasis (Dai et al. 2020; Lagraauw et al. 2015). Excessive or prolonged stress overactivates the hypothalamic–pituitary–adrenal (HPA) axis (Milligan et al. 2021), leading to physiological dysfunctions such as immune, neurochemical, and endocrine disorders (Torpy et al. 2007). As the center of metabolic activity, the liver is critical for maintaining synthetic and secretory functions and is highly sensitive to environmental stress (Liu et al. 2022). Chronic stress induces hepatic metabolic disorders and inflammatory accumulation, leading to liver injury and potentially severe outcomes such as hepatocellular carcinoma, which in turn exacerbates depressive symptoms (Qin et al. 2019; Shea et al. 2021). A study suggested that regulating hepatic metabolism can alleviate depression‐like behaviors through a negative feedback mechanism (Qin et al. 2019). In traditional Chinese medicine, “liver qi stagnation” caused by low mood or excessive psychological stress impairs liver function and spleen transportation, manifesting as symptoms such as chest/rib pain (liver qi stagnation) and nausea (spleen deficiency), indicating a close link between liver dysfunction and depression (Li et al. 2020). Thus, emotional regulation and liver protection are crucial for preventing stress‐induced depression in modern society.

Pyroptosis, a novel form of programmed cell death dependent on caspase‐1, is closely associated with inflammation driven by IL‐1β or IL‐18. The caspase‐1 mediated NF‐κB activation pathway is triggered by IL‐1 secretion (Sollberger et al. 2014). Caspase‐1, a classic inflammatory caspase, can prevent chronic restraint stress (CRS)‐induced depression‐like behaviors by regulating gut microbiota components (Li et al. 2023), and caspase‐1 deficiency has been shown to prevent depressive behavior in animal studies (Li et al. 2018).

Current clinical management of depression mainly relies on antidepressants such as selective serotonin reuptake inhibitors (SSRIs), tricyclic antidepressants (TCAs), monoamine oxidase inhibitors (MAOIs), and noradrenergic and specific serotonergic antidepressants (NaSSAs). However, these drugs often cause adverse effects (e.g., insomnia, diarrhea, headache), limiting their clinical application (Shelton 2019). These adverse effects limit the use of drugs in treating depression. Thus, there is a need to identify bioactive alternatives with fewer side effects. Green tea, consumed widely for millennia, has recently been shown to reduce depression‐like behavior in mice (Jia et al. 2023). Epigallocatechin‐3‐gallate (EGCG), the main component of green tea, exhibits multiple biological activities, including anti‐inflammatory, anti‐fibrotic, anti‐oxidative stress, anti‐apoptotic, autophagy‐promoting, and anti‐tumor effects (Zhang et al. 2023). Studies have indicated that cellular pyroptosis plays a key role in liver injury (Gan et al. 2022), and EGCG can reduce hepatocellular pyroptosis (Mao et al. 2024) and inhibit pyroptosis‐related factors to exert antidepressant effects (Abdelmeguid et al. 2022). Therefore, regulating cellular pyroptosis to prevent liver injury may be a potential target for improving depression.

In this study, we used the CUMS model (Abdelmeguid et al. 2022) to explore the effects of EGCG under chronic stress. Our objective was to verify whether EGCG alleviates liver injury and improves depression‐like behaviors in female rats by inhibiting the NF‐κB/caspase‐1 pyroptosis pathway.

## 2 Experimental Materials and Methods

### 2.1 Experimental Animals

Thirty‐three female and eight male SPF‐grade SD rats (6 weeks old, weighing 150 ± 10 g; license No. SCXK [Xiang] 2019–0004) were purchased from Hunan Lake Jingda Laboratory Animal Co. Ltd. Rats were housed in chambers with controlled temperature (25°C ± 2°C), humidity (60% ± 5%), and a 12‐h light/dark cycle, with free access to food and water. After 1 week of acclimation, the experiment was initiated. All animal procedures were approved by the Animal Ethics Committee of Guilin Medical University (approval No. GLMC20243265) and complied with current animal welfare guidelines.

### 2.2 Experimental Methods

#### 2.2.1 Model Establishment and Drug Administration

**FIGURE 1:**
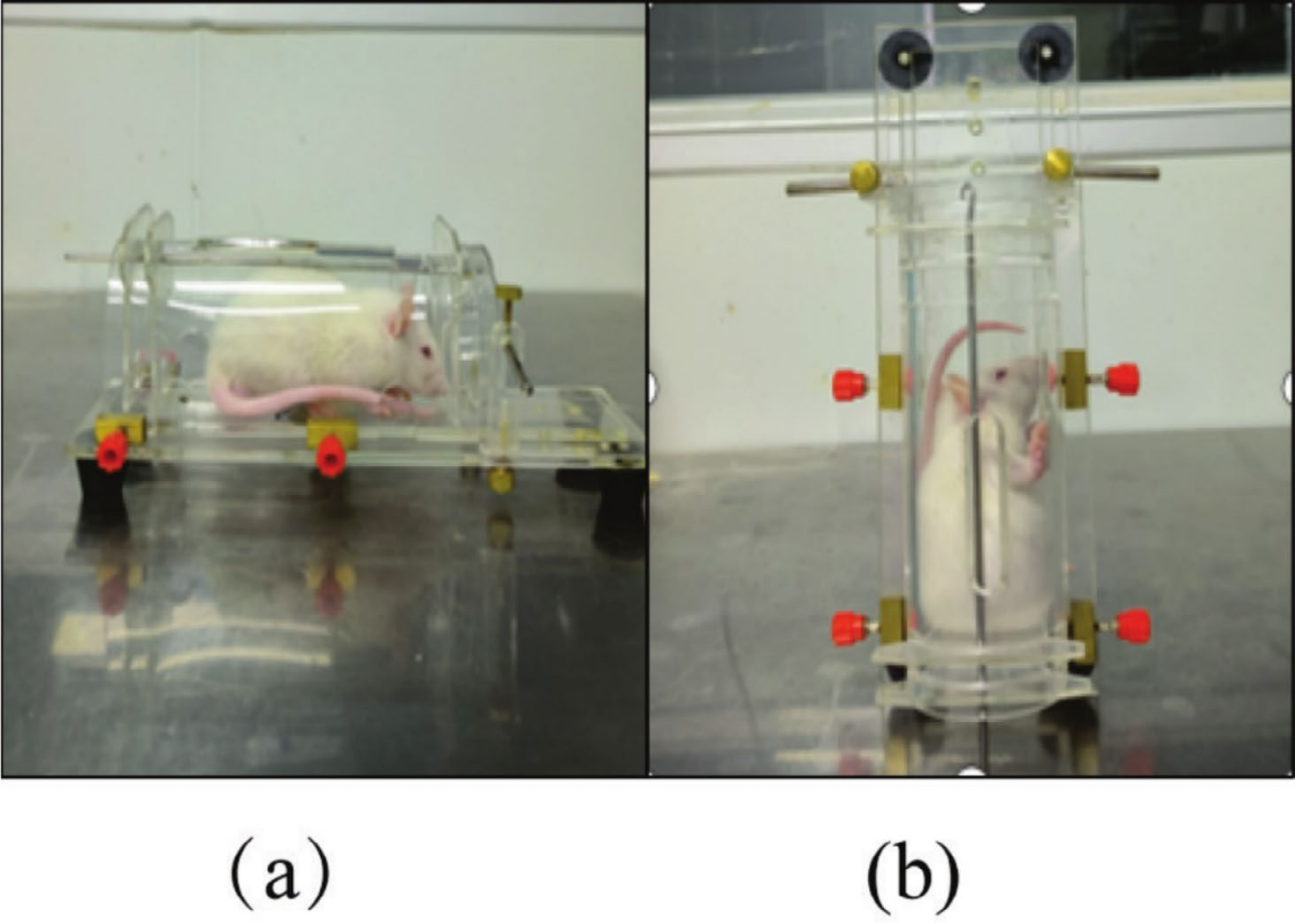
The pictures of restraint stress in rat: (a) is the diagram of horizontal binding stress in rats; (b) is the diagram of vertical binding stress in rats.

**FIGURE 2:**
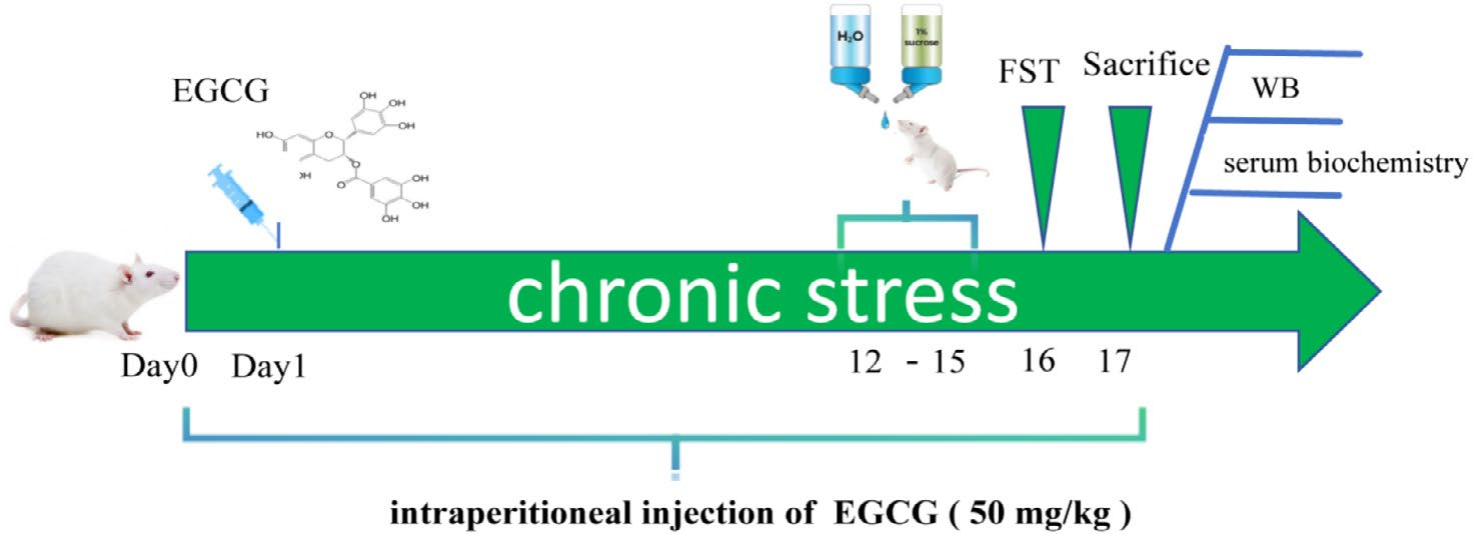
Study design and drug schedule.

After 1 week of acclimation, female SD rats were randomly divided into three groups: control group (cont, *n* = 9), stress group (str, *n* = 12), and EGCG intervention group (str + EGCG, *n* = 12). Male SD rats were assigned to the stress group (STR, *n* = 8). To avoid circadian rhythm interference, all groups except the control group were subjected to two random stimuli daily to establish the CUMS model (Kocot and Wroblewska 2022). Stimuli included: daily restraint stress (4 h, 9:00–13:00), overnight lighting, and wet bedding (6 h) for 2 weeks. Detailed weekly stress protocols are shown in Table 1, and restraint methods are illustrated in Figure 1. EGCG (No. HY‐13653, purity 99.30%; MCE Company) was dissolved in saline to a concentration of 50 mg/kg and administered intraperitoneally to the str + EGCG group 1 h before daily stress (0.5 mL/100 g body weight). The control and stress groups received equal volumes of sterile 0.9% saline. The experiment is detailed in Figure 2.

**TABLE 1:** Detailed stress mode of chronic unpredictable mild stress in rats.

Date (day)	Stress regimen		
1	Bound stress (vertical position)	4 h	Light overnight
2	Bound stress (horizontal placement)	4 h	Wet mat material
3	Bound stress (vertical position)	4 h	Light overnight
4	Bound stress (horizontal placement)	4 h	Wet mat material
5	Bound stress (vertical position)	4 h	Light overnight
6	Bound stress (horizontal placement)	4 h	Wet mat material
7	Bound stress (vertical position)	4 h	Have a rest

#### 2.2.2 Sucrose Preference Test (SPT)

Anhedonia, a core symptom of depression, is characterized by reduced responsiveness to pleasurable stimuli (Kong et al. 2024). SPT was performed on day 15 of chronic stress, following a 4‐day protocol: Day 1: Two bottles of 1% sucrose solution were provided for free access. Day 2: One bottle of 1% sucrose solution was replaced with pure water, with positions swapped at 12 h. Day 3: Food and water were deprived. Day 4: Formal test: one bottle of 1% sucrose solution and one bottle of pure water were provided for 24 h, with positions swapped every 12 h to avoid position preference. After 24 h, the consumption of sucrose solution and pure water was recorded, and the sucrose preference rate was calculated as follows (Zhang et al. 2024):

$$\text{Sucrose preference rate}\;\left(\%\right)=\left(\text{sucrose solution consumption}/\left[\text{sucrose solution consumption}+\text{pure water consumption}\right]\right)\times 100\%$$

#### 2.2.3 Forced Swimming Test (FST)

FST, also known as the “behavioral despair test,” evaluates rodents' responses to inescapable drowning threat, reflecting susceptibility to negative emotions (Raez et al. 2020). Immobility time is positively correlated with depression‐like behavior. Rats were individually placed in a transparent vertical plastic cylinder (2/3 filled with water at 25° ± 1°C; water depth prevented contact with the cylinder bottom). The 6‐min test included a 2‐min acclimation period, followed by recording immobility time (defined as minimal movement to maintain balance with head above water) for the remaining 4 min. After testing, rats were dried and returned to their cages, and water was replaced for subsequent tests.

#### 2.2.4 Biochemical Measurement of Serum Lipid and Liver Function Indicators

After behavioral tests, rats were fasted and deprived of water for 12 h. Under anesthesia with 4% pentobarbital, blood was collected from the heart, allowed to stand at room temperature for 2 h, and centrifuged at 8000 r/min for 10 min at 4°C to obtain serum, which was stored at −80°C. Serum levels of triglyceride (TG), total cholesterol (TC), high‐density lipoprotein cholesterol (HDL‐C), low‐density lipoprotein cholesterol (LDL‐C), alanine transaminase (ALT), and aspartate transaminase (AST) were measured using kits (Nanjing Jiancheng Bioengineering Institute) according to the manufacturer's instructions. Absorbance was read at 510 nm, and concentrations were calculated.

#### 2.2.5 Western Blot Analysis of Pyroptosis‐Related Proteins in Liver and Brain Tissues

Approximately 0.05 g of liver or brain tissue was homogenized in pre‐cooled protein lysis buffer, sonicated, and incubated on ice for 1 h to extract proteins. Protein concentration was determined using a BCA kit (P0010, Shanghai Biyuntian Biotechnology Co. Ltd.). Thirty micrograms of total protein were separated by 8%–12% SDS‐PAGE and transferred to PVDF membranes (Millipore, USA). Membranes were blocked with 3% skim milk for 1 h at room temperature, then incubated overnight at 4°C with primary antibodies: NF‐κB (Q04206), caspase‐1 (P29452), IL‐1β (P01584), IL‐18 (P70380), GAPDH (P04406), caspase‐8 (4790S; CST), caspase‐3 (ab184787; Abcam), cleaved‐caspase‐9 (P012889), cleaved‐caspase‐8 (M011932; Epizyme Biotech), cleaved‐caspase‐3 (68773–1‐Ig), Bax (50599–2‐Ig), Bcl2 (68103–1‐Ig; Proteintech), caspase‐9 (WL03421), and cleaved‐caspase‐1 (WL03450; Wanleibio). Membranes were then incubated with horseradish peroxidase (HRP)‐labeled secondary antibody (Anti‐Rabbit IgG [H + L], SA00001‐2) for 1 h at room temperature. Signals were detected using Super Signal West Femto chemiluminescent substrate (Lee Ji Bio), and band gray values were analyzed with an automatic gel imaging system (Protein Simple Inc., Devens, MA, USA).

#### 2.2.6 Statistical Analysis

Data were analyzed using GraphPad Prism 9.5.1 software and expressed as mean ± standard deviation. Differences were compared using one‐way ANOVA followed by Dunnett's post hoc test or unpaired Student's t‐test. A *p*‐value < 0.05 was considered statistically significant.

## 3 Experimental Results

### 3.1 EGCG Alleviates CUMS‐Induced Depression‐Like Behaviors in Rats

**FIGURE 3:**
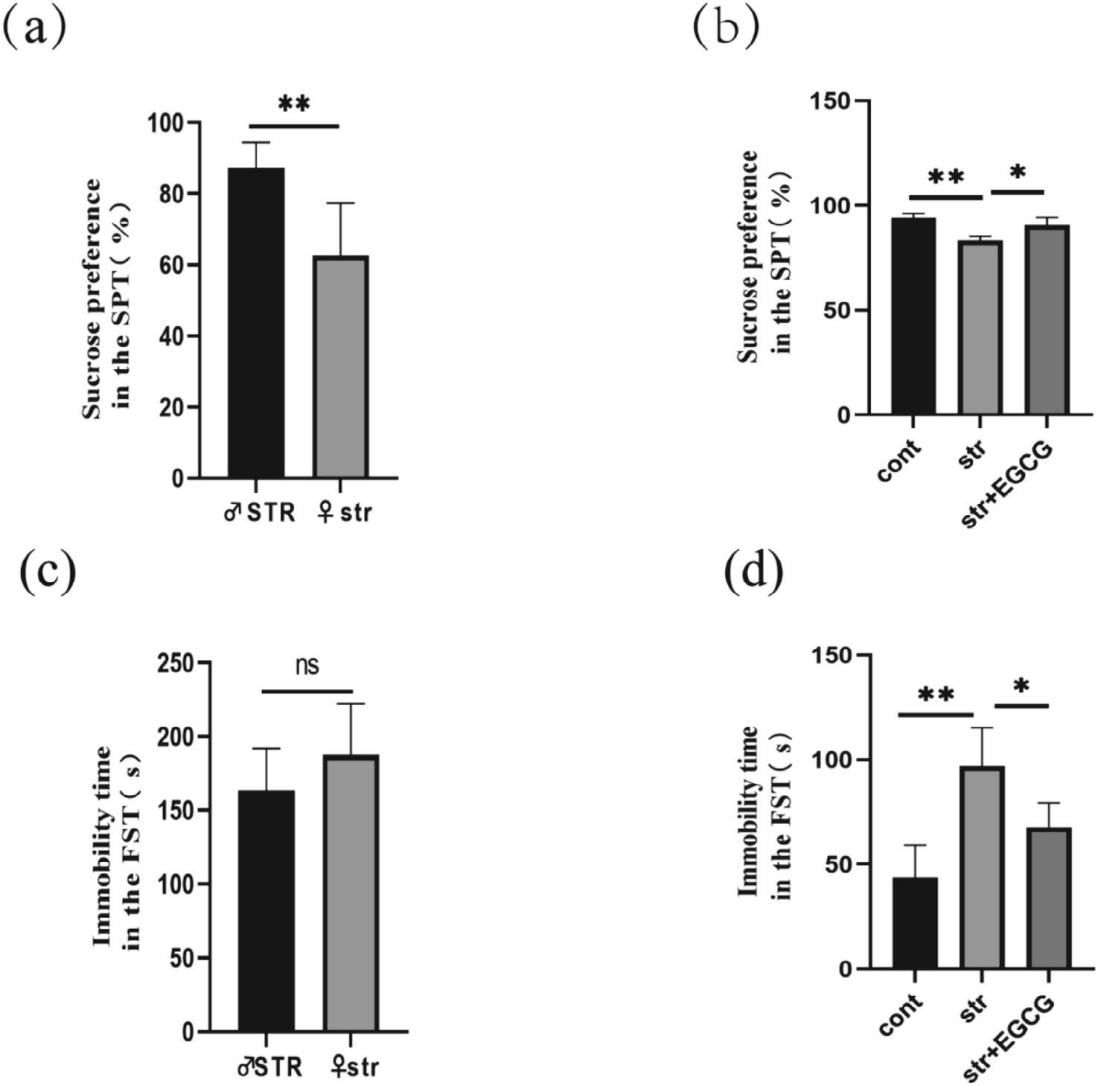
Effects of EGCG on depression‐like behaviors in rats. (a, b) Sucrose preference experiment (*n* = 6–8); (c, d) forced swimming experiment (*n* = 6–8). The cont, Control group; ns, not significant; str, Chronic stress group; str + EGCG, EGCG intervention group; STR, male chronic stress group. (ns): *p* > 0.05; (*): *p* < 0.05; (**): *p* < 0.01.

Reduced sucrose preference and increased FST immobility time are typical symptoms of depression in rats (Ma et al. 2021). After 2 weeks of CUMS, SPT and FST were performed to evaluate depression‐like behaviors (Figure 3). Female str rats showed significantly lower sucrose preference than male STR rats (*p* < 0.01; Figure 3a). Compared with the control group, sucrose preference was significantly reduced in the str group (*p* < 0.01), indicating anhedonia, while EGCG intervention (50 mg/kg) significantly reversed this reduction (*p* < 0.001; Figure 3b). Female str rats showed slightly more activity than male STR rats (Figure 3c). Immobility time was significantly longer in the str group than in the control group (*p* < 0.001), indicating behavioral despair, but EGCG intervention significantly shortened this time (*p* < 0.001; Figure 3d). These results suggest that EGCG can significantly alleviate CUMS‐induced depression‐like behaviors (anhedonia and behavioral despair) in rats.

### 3.2 EGCG Improves CUMS‐Induced Reduction in Body Weight and Liver Index

**FIGURE 4:**
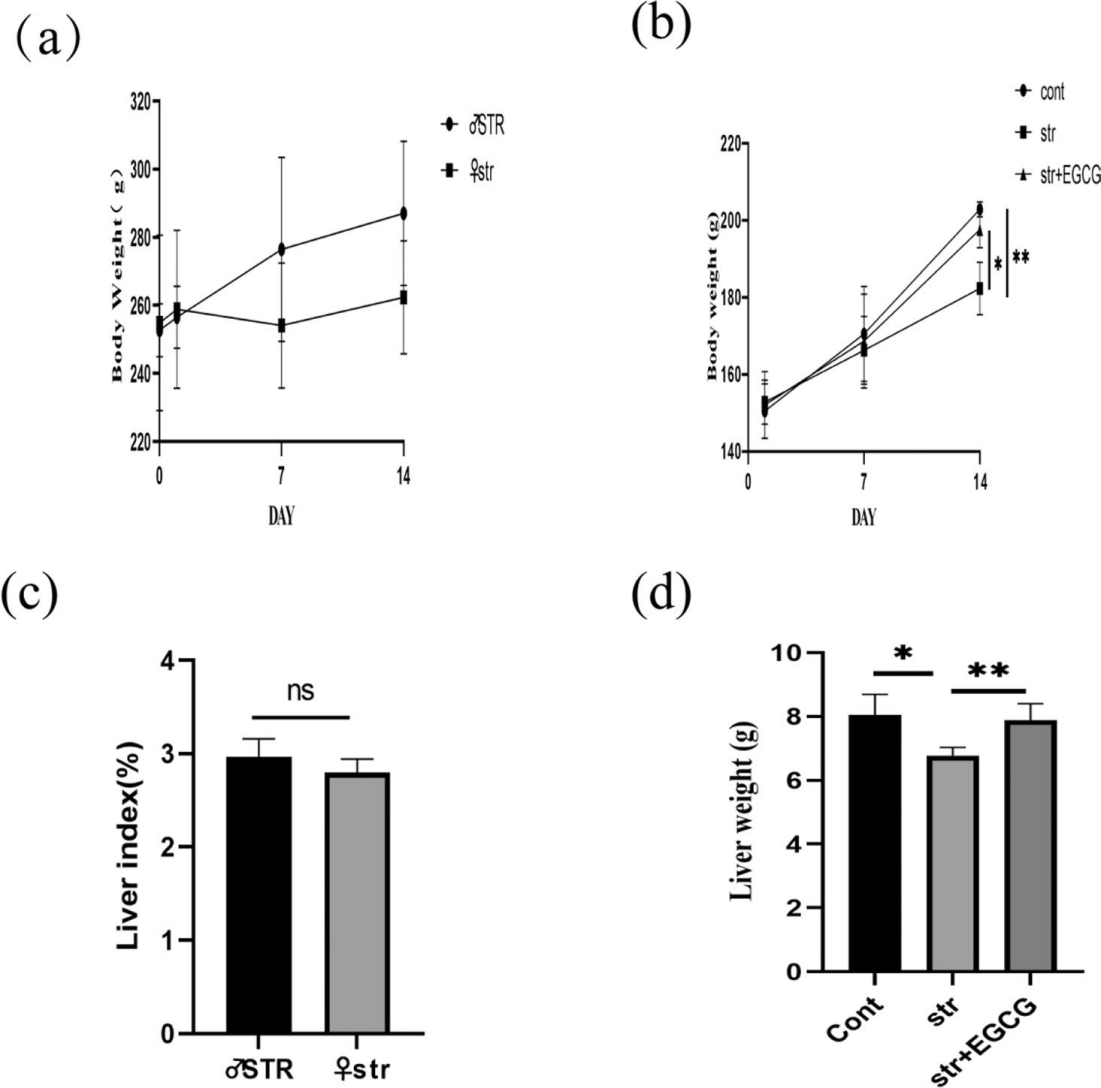
Effect of EGCG on body weight and liver index in chronic stress rats. (a, b) Trend of weight gain (*n* = 6); (c, d) liver weight (*n* = 6–8). The cont, control group; str, chronic stress group; str + EGCG, EGCG intervention group; STR, male chronic stress group. (ns): *p* > 0.05; (*): *p* < 0.05; (**): *p* < 0.01.

Weight loss is a typical symptom of depression, and EGCG alleviated CUMS‐induced reductions in body weight and liver index (Figure 4). Compared with male STR rats, female str rats showed more significant weight loss and a relatively higher liver index. The str group had significantly lower body weight (*p* < 0.05) and liver index (*p* < 0.01) than the control group, while EGCG intervention significantly mitigated these reductions.

### 3.3 EGCG Reverses CUMS‐Induced Serum Lipid and Liver Function Abnormalities

**FIGURE 5:**
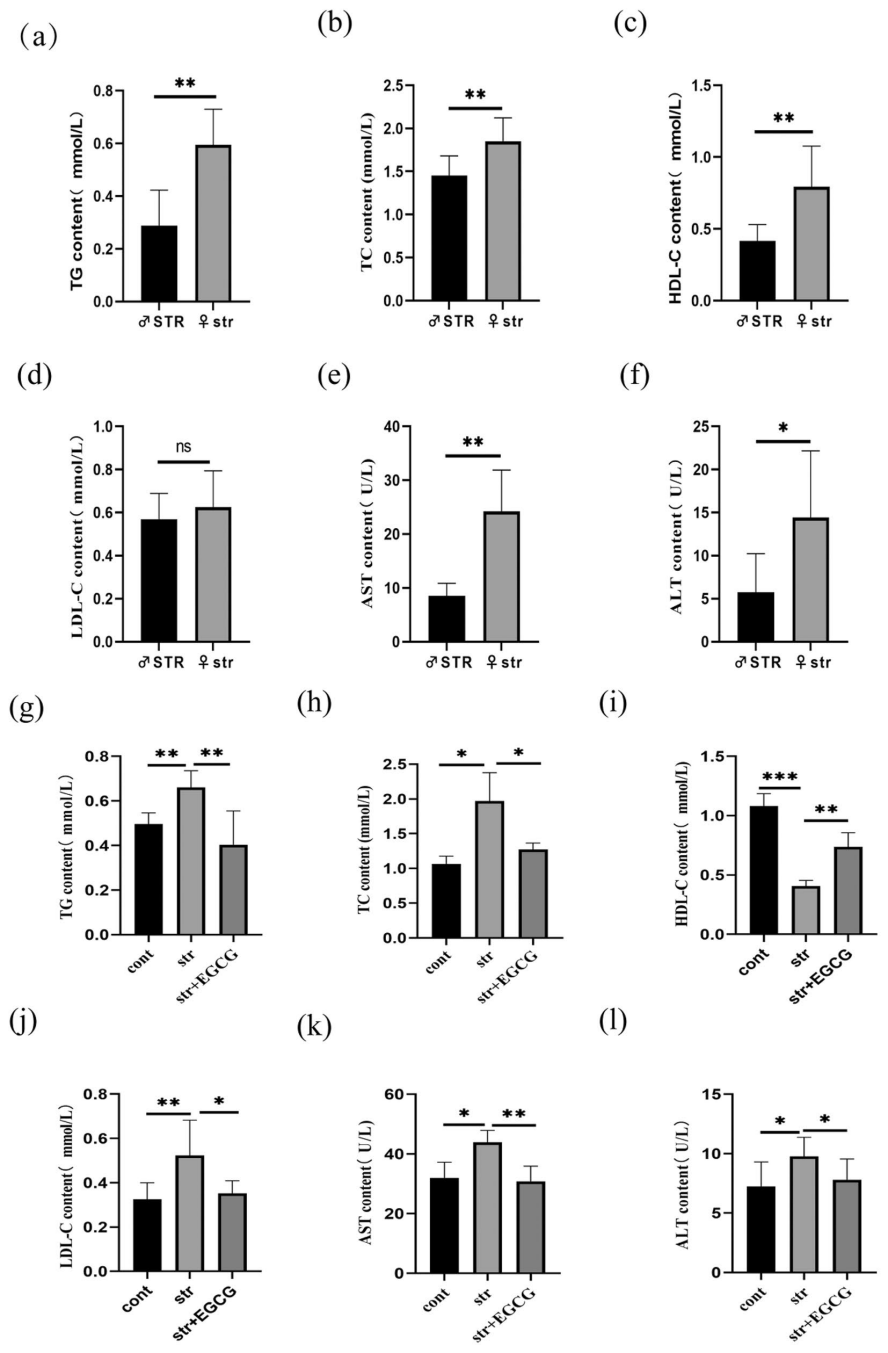
EGCG reversed CUMS‐mediated serum dyslipidemia in rats. (a–f) TG, TC, HDL‐C, LDL‐C, AST, and ALT content in females compared with SD rats in the male stress group (*n* = 6); (g–l) the contents of TG, TC, HDL‐C, LDL‐C, AST, and ALT between different groups of male and female SD rats (*n* = 6). The cont, control group; str, chronic stress group; str + EGCG, EGCG intervention group; STR, male chronic stress group. (ns): *p* > 0.05; (*): *p* < 0.05; (**): p < 0.01; (***): *p* < 0.001.

Blood lipids reflect metabolic status (Lee et al. 2022). As shown in Figure 5a–f, female str rats had significantly higher levels of TC (*p* < 0.01), TG (*p* < 0.01), and AST (*p* < 0.05) than male STR rats, suggesting greater sensitivity to CUMS‐induced lipid disorders. Compared with the control group, the str group showed significantly increased TC (*p* < 0.05), TG (*p* < 0.01), LDL‐C (*p* < 0.001), ALT (*p* < 0.05), and AST (*p* < 0.01), and decreased HDL‐C (*p* < 0.001; Figure 5g–l). EGCG intervention (50 mg/kg) reversed these abnormal changes.

### 3.4 EGCG Reduces Pyroptosis‐Related Protein Expression in the Liver of CUMS Rats

**FIGURE 6:**
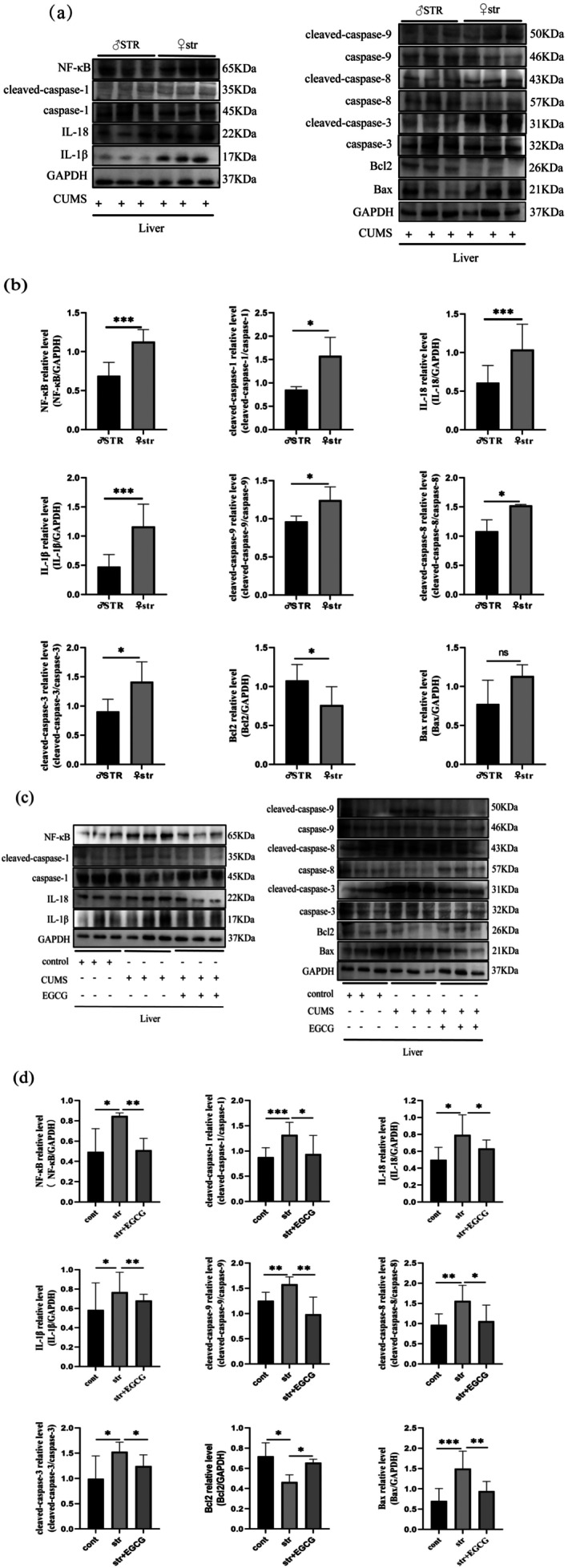
Effect of EGCG on the expression of NF‐κB, caspase‐1, and pyroptosis‐related proteins in rat liver tissues under chronic stress. Representative western blot images (a) and quantitative data (b) showing the levels of NF‐κB, cleaved‐caspase‐1, IL‐18, IL‐1β, cleaved‐caspase‐9, cleaved‐caspase‐8, cleaved‐caspase‐3, Bcl2, and Bax proteins in the liver tissues of male and female rats under chronic stress (*n* = 6–10). Representative western blot images (c) and quantitative data (d) illustrating the levels of NF‐κB, cleaved‐caspase‐1, IL‐18, IL‐1β, cleaved‐caspase‐9, cleaved‐caspase‐8, cleaved‐caspase‐3, Bcl2, and Bax proteins in the liver tissues of female rats of the control, stress, and EGCG groups (*n* = 6–10). The data are expressed as the mean ± SD, and the results were analyzed by Student's *t*‐test or one‐way ANOVA, followed by *post hoc* analysis. The cont, control group, CUMS, chronic unpredictable mild stress; str, chronic stress group; str + EGCG, EGCG intervention group; STR, male chronic stress group. (*): *p* < 0.05; (**): *p* < 0.01; (ns): *p* > 0.05.

NF‐κB signaling is activated in damaged hepatocytes (Wang et al. 2020) and is closely associated with caspase‐1, a key mediator of pyroptosis. Activated caspase‐1 further triggers IL‐1β and IL‐18 release, promoting inflammatory responses (Frank and Vince 2019). Western blot results (Figure 6) showed: Compared with male STR rats, female str rats had significantly upregulated liver expression of NF‐κB, IL‐1β, IL‐18, cleaved‐caspase‐1, cleaved‐caspase‐9, cleaved‐caspase‐8, cleaved‐caspase‐3, and Bax, and downregulated Bcl2. Compared with the control group, the str group showed activated hepatic NF‐κB pathway, significantly increased expression of cleaved‐caspase‐1, IL‐1β, IL‐18, cleaved‐caspase‐9, cleaved‐caspase‐8, cleaved‐caspase‐3, and Bax, and decreased Bcl2. EGCG intervention inhibited NF‐κB activation, significantly reduced the expression of cleaved‐caspase‐1, IL‐1β, cleaved‐caspase‐9, cleaved‐caspase‐8, cleaved‐caspase‐3, and Bax, and increased Bcl2. These results indicate that EGCG reduces the expression of hepatic pyroptosis‐related proteins and inhibits hepatic pyroptosis in CUMS rats.

### 3.5 EGCG Inhibits Pyroptosis‐Related Protein Expression in the Brain of CUMS Rats

**FIGURE 7:**
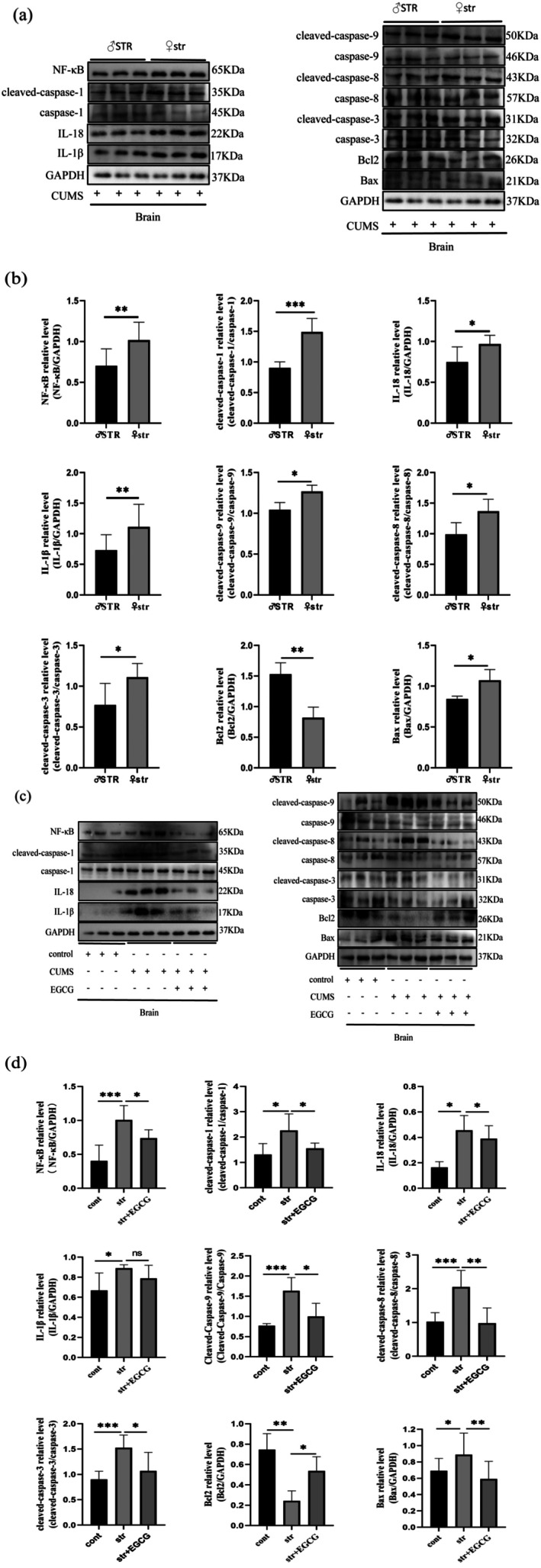
Effect of EGCG on the expression of pyroptosis‐related proteins in rat brain tissues under chronic stress. Representative western blot images (a) and quantitative data (b) showing the levels of NF‐κB, cleaved‐caspase‐1, IL‐18, IL‐1β, cleaved‐caspase‐9, cleaved‐caspase‐8, cleaved‐caspase‐3, Bcl2, and Bax proteins in the brain tissues of male and female rats under chronic stress (*n* = 6–10). Representative western blot images (c) and quantitative data (d) illustrating the levels of NF‐κB, cleaved‐caspase‐1, IL‐18, IL‐1β, cleaved‐caspase‐9, cleaved‐caspase‐8, cleaved‐caspase‐3, Bcl2, and Bax proteins in the brain tissues of female rats of the control, stress, and EGCG groups (*n* = 6–10). The data are expressed as the mean ± SD, and the results were analyzed by Student's *t*‐test or one‐way ANOVA, followed by *post hoc* analysis. The cont, control group; CUMS, chronic unpredictable mild stress; str, chronic stress group; str + EGCG, EGCG intervention group; STR, male chronic stress group. (*): *p* < 0.05; (**): *p* < 0.01; (***): *p* < 0.001; (ns): *p* > 0.05.

To explore whether EGCG acts through the liver‐brain axis, pyroptosis‐related proteins in brain tissue were analyzed (Figure 7). Consistent with liver findings: Female str rats showed significantly higher brain expression of NF‐κB, cleaved‐caspase‐1, IL‐1β, IL‐18, cleaved‐caspase‐9, cleaved‐caspase‐8, cleaved‐caspase‐3, and Bax, and lower Bcl2 than male STR rats. Compared with the control group, the str group had elevated levels of these proteins (except Bcl2), which were reversed by EGCG intervention. These results suggest that EGCG inhibits pyroptosis‐related protein expression in both liver and brain tissues of CUMS rats, with more pronounced effects in females.

## 4 Discussion

Stress is closely linked to disease risk: moderate stress can stimulate bodily potential, but excessive, chronic, uncontrollable stress is a risk factor for chronic non‐communicable diseases, consistent with the social‐psycho‐physiological model (Agorastos and Chrousos 2022; Song and Kim 2021). Chronic stress induces hepatic chronic inflammatory damage and collagen deposition around central veins (Corona‐Perez et al. 2017), activates the autonomic nervous system and HPA axis, and sustained HPA hyperactivation with elevated cortisol levels contributes to depression (Tafet and Nemeroff 2016). Clinically, depression is characterized by fatigue, social impairment, and anhedonia (Rahim and Rashid 2017). Liao et al. reported that CUMS rats exhibit anhedonia and learned helplessness, a gold standard for successful depression modeling (Liao et al. 2023), consistent with our results. Restraint stress mimics the pathogenesis of human psychosomatic diseases, making it a widely used model for studying physical and mental disorders (Yang, Wang, et al. 2024; Yang, Frolinger, et al. 2024). Thus, we used a CUMS model to explore stress‐induced mood disorders.

Due to physiological and social role differences, men and women respond differently to stress: women are more sensitive to life stress, with higher depression susceptibility and more severe outcomes (Cohen et al. 2019). The China Mental Health Survey (CMHS) showed higher depression prevalence in women (lifetime OR = 1.44; 12‐month OR = 1.41) (Lu et al. 2021), with increased risks of suicidal behavior and liver disease (Kim et al. 2023; Xu et al. 2021). Animal studies found that both sexes develop depression‐like behaviors after chronic stress, but only females show persistence (Yang, Wang, et al. 2024; Yang, Frolinger, et al. 2024), and female rats exhibit altitude‐dependent increases in depression‐like behaviors not observed in males (Sheth et al. 2018). Thus, we focused on female rats, with male rats as controls.

Chronic stress is a risk factor for depression and anxiety, with anhedonia and learned helplessness as core symptoms (Tang et al. 2021). SPT evaluates motivation and emotional states via sweetness preference, while FST reflects “behavioral despair” through immobility in inescapable water (Hodes et al. 2015). Female rodents are more susceptible to unpredictable stress: 6 days of varied stress induce anxiety/depression‐like behaviors in females but not males (Kokras et al. 2015), facilitating research on sex‐specific stress responses. A study on Wistar rats showed higher FST immobility in females, which is alleviated by antidepressants (Fonseca‐Rodrigues et al. 2022), while baseline sucrose preference shows no sex difference (Bian et al. 2023). Our results showed lower sucrose preference and longer FST immobility in female str rats than in male STR rats, confirming more severe CUMS‐induced depression‐like behavior in females. EGCG intervention alleviated these changes, indicating its potential to mitigate stress‐induced anhedonia and despair.

Chronic stress disrupts metabolic homeostasis, causing weight loss (Moran and Delville 2024), and HPA axis hyperactivation, which impairs liver function and triggers inflammation (Barloese et al. 2020). A study found that CCl4‐induced liver index and weight increases in female mice but not males (Zhu et al. 2023). Our results showed reduced liver index and weight in female str rats (vs. controls), which were reversed by EGCG (50 mg/kg), suggesting EGCG alleviates stress‐induced hepatic inflammatory progression by improving liver index and weight.

Abnormal blood lipids are linked to depression and suicide (Oh and Kim 2017; Wang et al. 2020). Depressed patients have elevated TC, TG, and LDL‐C, with antidepressants (e.g., fluoxetine) improving symptoms and LDL‐C/HDL‐C ratios (Zorkina et al. 2024). Chinese children/adolescents with major depressive disorder (MDD) show similar lipid abnormalities (Liu et al. 2024), and female depressed patients are more prone to dyslipidemia during treatment (Yang et al. 2022). Chronic stress also elevates liver function indicators and inflammatory factors (Yang et al. 2022). Our results showed more severe dyslipidemia and liver injury in female str rats than in male STR rats, with EGCG reversing CUMS‐induced lipid abnormalities, consistent with our previous study (Zhang et al. 2024). These findings confirm that chronic stress induces depression‐like behaviors and liver injury (reduced liver index, elevated AST/ALT), alleviated by EGCG.

Pyroptosis plays a key role in chronic liver injury, liver fibrosis, and hepatocellular carcinoma (Gan et al. 2022; Zou et al. 2023). It is characterized by membrane pore formation, rupture, swelling, and release of IL‐1β/IL‐18, triggering inflammation and immune responses (Rao et al. 2022; Zou et al. 2023). NF‐κB activation upregulates NLRP3 inflammasome transcription; NLRP3 binds caspase‐1 via ASC, activating caspase‐1 (Fu and Wu 2023). Activated caspase‐1 cleaves GSDMD to induce pyroptosis and processes pro‐IL‐1β/pro‐IL‐18 into mature forms, promoting chronic inflammation (You et al. 2022). Recent studies showed that EGCG prevents nonalcoholic fatty liver disease via the NLRP3/caspase‐1/GSDMD pathway (Mao et al. 2024), but its role in caspase‐1‐mediated pyroptosis and depression remains unclear. Our study focused on NF‐κB activation and its association with caspase‐1‐related pyroptosis factors, finding that CUMS activates the hepatic NF‐κB/caspase‐1 pathway, increasing IL‐1β/IL‐18 release and upregulating apoptotic factors (cleaved‐caspase‐9/8/3, Bax) while downregulating Bcl2, with more pronounced effects in females. EGCG inhibited these responses, reducing pyroptosis‐induced inflammation and liver injury.

Most depression studies focus on direct neural effects of drugs. For example, total paeony glycosides inhibit hippocampal neuronal apoptosis and reduce CUMS‐induced caspase‐1/IL‐1β elevation (Su et al. 2024), and lycopene alleviates hippocampal microglial pyroptosis via the cathepsin B/NLRP3 pathway (Zhu et al. 2023). EGCG affects brain NF‐κB (Zhou et al. 2018), and NF‐κB activation is linked to downstream caspase‐1 activation. To explore liver‐brain axis involvement, we analyzed brain tissues: CUMS induced high expression of cleaved‐caspase‐1, inflammatory factors, and apoptotic factors in rat brains (more pronounced in females), which was reduced by EGCG. These findings suggest EGCG may improve depression‐like behavior by inhibiting NF‐κB, reducing hepatic pyroptosis/liver injury, and lowering brain pyroptosis‐related factors, though the precise mechanism requires further study.

## 5 Conclusion

EGCG alleviates CUMS‐induced activation of the NF‐κB signaling pathway and pyroptosis in rat liver and brain tissues, reducing liver injury and improving blood lipid abnormalities, thereby ameliorating depression‐like behaviors. This study provides novel evidence that EGCG mitigates chronic stress‐induced physiological damage, supporting its potential as a therapeutic agent for preventing and managing stress‐induced depression. These findings also lay a mechanistic foundation for exploring EGCG as a safe and effective adjuvant therapy in clinical practice.

## Author Contributions

**Guixian Wu:** conceptualization (equal), data curation (equal), formal analysis (equal), investigation (equal), methodology (equal), visualization (equal), writing – original draft (equal), writing – review and editing (equal). **Jieyu Xu:** data curation (equal), resources (equal), writing – review and editing (equal). **Wanhua Wu:** data curation (equal), formal analysis (equal), investigation (equal), visualization (equal), writing – review and editing (equal). **Jian Jin:** data curation (equal), resources (equal), writing – review and editing (equal). **Shanqian Li:** data curation (equal), formal analysis (equal), investigation (equal), visualization (equal), writing – review and editing (equal). **Yue Huang:** funding acquisition (equal), resources (equal), writing – review and editing (equal). **Zongyi Zhang:** data curation (equal), formal analysis (equal), investigation (equal), visualization (equal), writing – review and editing (equal). **Yue Hu:** data curation (equal), formal analysis (equal), investigation (equal), visualization (equal), writing – review and editing (equal). **Yulin Zhang:** data curation (equal), formal analysis (equal), investigation (equal), visualization (equal), writing – review and editing (equal). **Yuling Liu:** data curation (equal), formal analysis (equal), investigation (equal), visualization (equal), writing – review and editing (equal). **Yao Xu:** data curation (equal), formal analysis (equal), investigation (equal), visualization (equal), writing – review and editing (equal). **Meimei Zheng:** data curation (equal), investigation (equal), visualization (equal), writing – review and editing (equal). **Chaozhi Xu:** data curation (equal), formal analysis (equal), investigation (equal), visualization (equal), writing – review and editing (equal). **Wenjun Lu:** resources (equal), writing – review and editing (equal). **Hongxian Wu:** conceptualization (equal), funding acquisition (equal), methodology (equal), project administration (equal), supervision (equal), writing – review and editing (equal). **Lina Hu:** conceptualization (equal), funding acquisition (equal), methodology (equal), project administration (equal), resources (equal), supervision (equal), writing – review and editing (equal).

## Conflicts of Interest

The authors declare no conflicts of interest.

## Data Availability

The authors will supply the relevant data in response to reasonable requests.
